# Development of a Web-Based Formative Self-Assessment Tool for Physicians to Practice Breaking Bad News (BRADNET)

**DOI:** 10.2196/mededu.9551

**Published:** 2018-07-19

**Authors:** Anne-Christine Rat, Laetitia Ricci, Francis Guillemin, Camille Ricatte, Manon Pongy, Rachel Vieux, Elisabeth Spitz, Laurent Muller

**Affiliations:** ^1^ EA 4360 APEMAC Université de Lorraine Nancy France; ^2^ Rheumatology Nancy University Hospital Nancy France; ^3^ CIC 1433 Clinical Epidemiology INSERM Nancy University Hospital Nancy France; ^4^ Equipe de psychologie de la santé de Metz EA 4360 APEMAC Université de Lorraine Metz France; ^5^ Department of Pediatrics Besançon University Besançon France

**Keywords:** bad news disclosure, health communication, physician-patient relationship, distance e-learning

## Abstract

**Background:**

Although most physicians in medical settings have to deliver bad news, the skills of delivering bad news to patients have been given insufficient attention. Delivering bad news is a complex communication task that includes verbal and nonverbal skills, the ability to recognize and respond to patients’ emotions and the importance of considering the patient’s environment such as culture and social status. How bad news is delivered can have consequences that may affect patients, sometimes over the long term.

**Objective:**

This project aimed to develop a Web-based formative self-assessment tool for physicians to practice delivering bad news to minimize the deleterious effects of poor way of breaking bad news about a disease, whatever the disease.

**Methods:**

BReaking bAD NEws Tool (BRADNET) items were developed by reviewing existing protocols and recommendations for delivering bad news. We also examined instruments for assessing patient-physician communications and conducted semistructured interviews with patients and physicians. From this step, we selected specific themes and then pooled these themes before consensus was achieved on a good practices communication framework list. Items were then created from this list. To ensure that physicians found BRADNET acceptable, understandable, and relevant to their patients’ condition, the tool was refined by a working group of clinicians familiar with delivering bad news. The think-aloud approach was used to explore the impact of the items and messages and why and how these messages could change physicians’ relations with patients or how to deliver bad news. Finally, formative self-assessment sessions were constructed according to a double perspective of progression: a chronological progression of the disclosure of the bad news and the growing difficulty of items (difficulty concerning the expected level of self-reflection).

**Results:**

The good practices communication framework list comprised 70 specific issues related to breaking bad news pooled into 8 main domains: opening, preparing for the delivery of bad news, communication techniques, consultation content, attention, physician emotional management, shared decision making, and the relationship between the physician and the medical team. After constructing the items from this list, the items were extensively refined to make them more useful to the target audience, and one item was added. BRADNET contains 71 items, each including a question, response options, and a corresponding message, which were divided into 8 domains and assessed with 12 self-assessment sessions. The BRADNET Web-based platform was developed according to the cognitive load theory and the cognitive theory of multimedia learning.

**Conclusions:**

The objective of this Web-based assessment tool was to create a “space” for reflection. It contained items leading to self-reflection and messages that introduced recommended communication behaviors. Our approach was innovative as it provided an inexpensive distance-learning self-assessment tool that was manageable and less time-consuming for physicians with often overwhelming schedules.

## Introduction

Bad news is defined as any information that adversely and seriously affects an individual's view of their future [1]. Any physician will have to deliver bad news during the course of their practice, but they are not always prepared to face these difficult situations. Learning how to deliver bad news is a real need for healthcare professionals because how bad news is relayed can have consequences that will affect the patient for a long time [2]. Patients interviewed regarding the delivery of bad news highlighted the importance of the relational dimension and wished for more time during the consultation and a multidisciplinary approach [3]. Appropriate communication behaviors during medical consultations have been shown to improve physician-patient relationships and alleviate patient anxiety [4-6].

Recommendations for delivering bad news have mainly been developed to deliver a diagnosis of cancer [7-11]. The protocol Setting-up, Perception, Invitation, Knowledge, Emotion, Strategy (SPIKES), in 6 steps, is the most well-known tool for delivering bad news to patients with cancer [11][11]. Guidelines in these models are straightforward and practical (ie, do not speak with medical jargon, manage time constraints and interruptions, determine the patient’s knowledge and expectations, deliver information in a progressive manner, check the patient’s understanding, try to maintain the patient’s hope, etc).

Although these guidelines were mainly developed in the context of cancer, every chronic disease, irreversible condition, or striking event can have a major effect on a patient’s life. For example, the bad news may relate to neurological, musculoskeletal, cardiac, renal, or respiratory disease or a serious congenital condition in a child.

Furthermore, publishing recommendations are not sufficient to implement and change behavior, if needed. Healthcare providers also need to develop adaptive abilities to manage the complexity of clinical situations in which each patient is unique. Therefore, training on how to deliver bad news helps physicians (1) to analyze their practice and difficulties; (2) to grasp the specific elements of the situation, including the context and individual characteristics of the patient; (3) to perceive the factors that influence physician-patient interactions (verbal and nonverbal attitudes); (4) to facilitate the development of a repertoire of communication strategies for delivering bad news that the patient finds difficult to accept (both cognitively and emotionally); and (5) to decipher and understand the patient’s emotional, cognitive, and behavioral responses in order to respond appropriately. Such training sessions should be provided both as initial and in-service training to build on the practice and experience of the physician.

In France, training sessions on how to deliver bad news, either as initial training or lifelong training, are not widespread [12]. To date, only motivated physicians have participated in training sessions on delivering bad news, even though student training is progressively increasing. Web-based technologies for delivering training could reach a greater number of physicians who cite high workloads, time pressures, and weak motivation, preventing them from engaging in intensive face-to-face training. Moreover, face-to-face training requires trained instructors, recognized for their experience in the field of delivering bad news, with mastery of medical, as well as psychological, social, and educational questions. A sound basis of evidence exists to support the use of computer-based techniques to improve general communication skills [13].

Over the past two decades, evidence from the literature indicates that formative self-assessment is an interesting way to improve learning (even “a *sine qua non* for effective learning” according to Black and William [14]) and is a necessary step for self-related learning. Indeed, with self-assessment, people reflect on their practice and their assessment of these practices and use their ratings to improve their skills [15]. Moreover, self-assessment plays a key role in the development of metacognitive skills. According to the research on self-assessment, learners who are skilled in metacognitive self-assessment and therefore aware of their abilities are more strategic and perform better than those who are unaware [16]. In terms of metacognitive knowledge, the accuracy of self-knowledge (ie, having accurate perceptions and making accurate judgments about one’s knowledge and skills) is relevant to learning [17]. However, the effectiveness of self-assessment depends to a large extent on the quality of the feedback provided to the learner [18]. Generally, feedback is an inherent catalyst for all self-regulated activities and contributes to guiding cognitive activities during which knowledge is acquired, fine-tuned, and restructured. External feedback should be used to reinforce internal feedback and be oriented toward formative assessment rather than summative assessment. In fact, the objective of feedback is to help the learner identify their strengths and weakness and to target areas that need work and improvement. Feedback should be provided using the principles of comprehensive and benevolent communication and should support an individual’s autonomy.

This strategy of learning also seems relevant for lifelong education because the learner can then directly integrate the self-assessment reflective process into their practice and thus proceed through an iterative process (but led by personal reflection).

The goal of our research project was to build a Web-based formative self-assessment tool for physicians to practice delivering bad news: the BReaking bAD NEws Tool (BRADNET). BRADNET was envisioned as a way to create a space for self-reflection and for self-practice analysis to minimize the deleterious effects of a poor way of breaking bad news about a disease, whatever the disease.

## Methods

**Figure 1:**
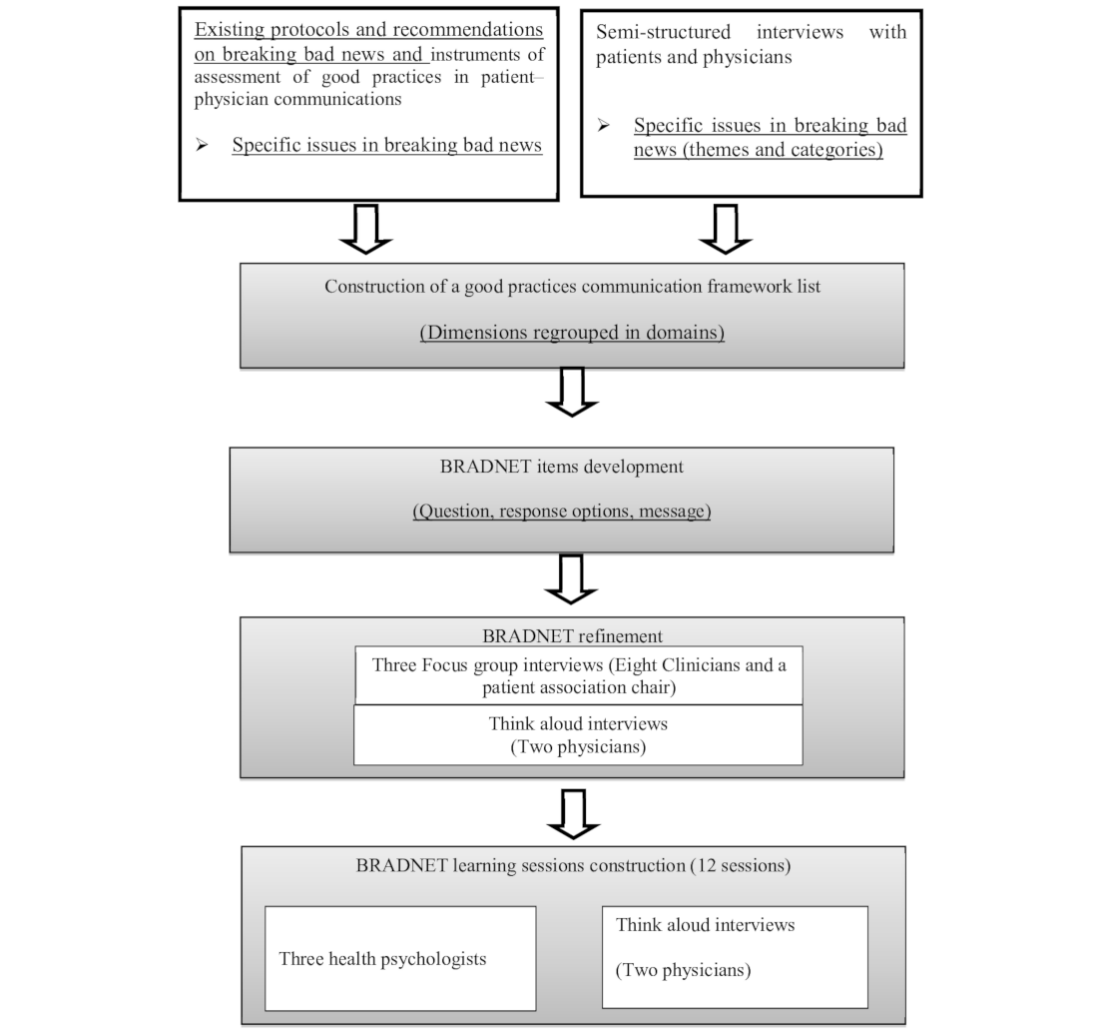
BRADNET development.

Development of and evidence-based health interventions are increasingly based on intervention mapping (IM), a protocol that consists of an iterative process integrating theoretical aspects, expert input and several data from the target population [19,20]. We followed the methodological line adopted by IM protocol, which consisted of a review of the literature, interviewing stakeholders, building a framework, and refining (Figure 1).

### Existing Protocols and Recommendations

We reviewed the existing protocols and recommendations for delivering bad news and instruments to assess patient-physician communication.

#### Semistructured Interviews With Patients and Physicians

Interview guides (one targeting patients and the second physicians) were the product of brainstorming with 3 health psychologists (LM, CR, ES) and 3 physicians (1 pediatrician, RV and 2 rheumatologists, FG and ACR) who were experienced in delivering bad news. Individual interviews were conducted by 6 trained psychologist interviewers, with 25 patients (11 with rheumatoid arthritis, 11 with heart failure, and 3 with cancer) and 22 physicians (10 nephrologists, 4 cardiologists, 4 oncologists, 3 neurologists, and 1 rheumatologist).

We used a referral sampling strategy in which clinicians from the research team recruited other physicians in private practice or hospitals. The sampling strategy was based on the maximum variation strategy to achieve maximum diversity on relevant aspects of breaking bad news [21,22]. Clinicians from the research team (rheumatology, cardiology, and oncology service) identified the patients in their clinical practice according to maximized variability criteria, as specified by the project team. Interviews took place in private practice or in hospitals, depending on the place of recruitment. The resultant 47 interviews were recorded and transcribed to fully capture the contents of each interview.

#### Thematic Analysis

Data analysis proceeded in an iterative manner using the grounded theory methods approach [23]. Two researchers (LM, ES) created codebooks and categories from inductive data, not preconceived hypotheses on which data might be overlaid [23,24]. Ten sessions with 2 health psychologists (LM, ES) were organized for constructing the 2 thematic codebooks, 1 for patients and 1 for physicians. Transcripts were double-coded by 2 analysts to ensure consistency [25]. The 2 coders (MMo, CR) were a psychologist and a PhD student. Data analyses were performed using NVivo v10 (QSR International, Melbourne, Australia). Coding discrepancies were resolved between the 2 coders by discussion on meaning.

#### Construction of the Good Practices Communication Framework List by Experts

Specific issues related to breaking bad news were retrieved from existing protocols. We also used recommendations for delivering bad news and instruments that assess patient-physician communication, and specific issues related to breaking bad news were retrieved from physician and patient interviews. Issues were then selected, and close issues were merged by 4 independent groups composed of 3 health psychologists (LM, CR, ES), 2 sociologists, 2 epidemiologists, and 3 clinicians (FG, ACR, RV). Finally, the 4 lists were pooled and restructured to achieve a consensus on a common list (good practices communication framework list) of dimensions to serve as a basis for item development. The dimensions were also grouped into domains.

#### BRADNET Item Development

**Figure 2:**
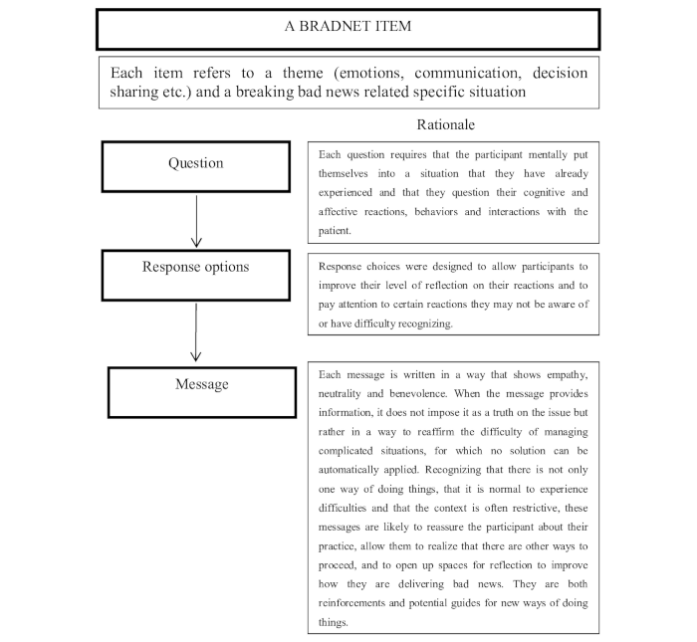
Format of a BRADNET item.

Items were then created from the good practices communication framework list (Figure 2). Their content was developed by a working group of 2 clinicians (ACR, RV) and 4 health psychologists (LM, CR, LR, ES). A BRADNET item was presented in the shape of a question that called for a reflection on one’s medical communication approach, response options, and a message that opened up perspectives.

Each item targeted a specific aspect of bad news delivery (such as the physician’s reaction to a patient’s anger). For each item, the question required the users to actively recall their past experience and beliefs and how they coped with that situation. The answering modalities ask respondents to position themselves according to various behaviors or attitudes of their practice (multiple responses were possible and no answer modality was considered a *good* or *bad* answer). Then, the message recognized the difficulty of the situation and the need for a personalized response in each situation. The system questioned the physician about the patient's needs and cognitive and emotional states, modified by a potentially stressful situation, and offered some clues to better account for the needs of the patient in this situation. The messages were designed to be benevolent (to identify possible difficulties and open to improvements), short (to avoid wasting any time), practical (practical applications, nontheoretical), and easy to apply.

Because BRADNET did not assess knowledge, the message was not personalized. Instead, it aimed to develop the understanding and awareness of cognitive-behavioral processes and the abilities and motivation necessary to improve skills related to breaking bad news. Even if the message was the same for everybody, how users received and processed the messages was different and could have different impacts. This may increase physician awareness of the importance of select behavioral or communication issues, validate some practices and thoughts, or introduce a reflection on the benefits of change. There was no score because BRADNET did not assess the level of knowledge or desirability. Questions were not intended for self-testing, but rather for reflection.

#### BRADNET Refinement: Focus Group Interviews

To ensure that physicians found BRADNET acceptable, understandable, and relevant to their patients’ condition, the tool was refined by a working group of the “Patient Therapeutic Education in Rheumatology Section” consisting of 8 clinicians familiar with delivering bad news, as well as experts in therapeutic education, and the chair of a patient association. Items and messages were reworded and completed to improve acceptability and content during 3 sessions of 2-3 hours each and during reviews and email exchanges. Items were also refined by 4 health psychologists (LM, CR, LR, and ES).

### Think-Aloud Interviews

“Think aloud” is a method for modeling cognitive processes. Different studies involving the think-aloud method agree that “this is a very direct method to gain insight into the knowledge and methods of problem solving in humans” [26]. The purpose of this method is to have easier access to thought processes and paths during the execution of a task and thus to collect qualitative data at the moment when tasks arise.

As part of the BRADNET development, the think-aloud approach was used to explore the impact of the items and messages and why and how these messages could change physicians’ relations with patients or how to deliver bad news. The objective was to determine whether the items could be useful to physicians, including initiating participant reflection on the meaning of the item, in terms of their own practices. The interviews were conducted with 2 physicians (neonatology and palliative care). Each physician spoke aloud during 3 sessions of 2 hours each.

#### BRADNET Learning Sessions Construction

Acquisition of knowledge increases when learning sessions are separated [27-29]. This separation is even more important when learning includes self-reflection and behavior modification. LM, CR, and ES organized the BRADNET items into formative self-assessment sessions according to the double perspective of progression: a chronological progression of the disclosure of the bad news and the growing difficulty of items (difficulty concerning the expected level of self-reflection). The aim was to include items relating to several domains in each self-training session. This format, including the short sessions, met two objectives: (1) reducing physicians’ time and (2) preventing cognitive overload and optimizing learning retention [30].

#### BRADNET Web-Based Platform Development: Theoretical Learning Principles

BRADNET was developed according to the cognitive load theory [28,30] and the cognitive theory of multimedia learning, which are based on 3 assumptions: the dual channel assumption (people possess separate processing systems for visual and verbal representations), the limited capacity assumption (the amount of processing is limited), and the active learning assumption (cognitive processing includes studying relevant incoming data, organizing this data into verbal and pictorial representations, and integrating these representations with each other and with prior knowledge [31]).

We envisioned BRADNET as a tool comprising static illustrations and printed text. Indeed, as compared with dynamic animation and narration learning formats, static illustrations and printed text have the advantage of reducing extraneous processing (cognitive processing that confounds training objectives), helping to manage intrinsic processing (cognitive processing of the key material) because students can control the pace of presentation and promoting learning retention. A static format allows people to control the pace and order of presentation and to engage in deeper processing such as making connections between words and pictures. It also encourages them to focus on the most relevant information. Finally, it allows for engaging people in active processing through activities such as generating explanations or answering questions during learning. A static format is also more appropriate than a dynamic one because BRADNET is a formative self-assessment tool, with items that lead to self-reflection. Static media can lead to better learning than dynamic media (such as animation and narration) [31]. Several multimedia instruction messages, consistent with how people learn will be used to enhance learning outcomes [32].

##### Multimedia Principle

The presentation of words and pictures, rather than words alone, causes learners to use both visual and verbal channels.

##### Reduce Extraneous Processing

- Coherence principle: exclusion of words or images that are not relevant
- Spatial contiguity principle: corresponding words and pictures are presented near to, rather than far from, each other on the screen to help build connections
- Temporal contiguity principle: learners must have corresponding words and images in working memory at the same time in order to make connections between them
- Signaling principle: cues highlighting the organization of the essential material designed to call attention to the important material

##### Manage Essential Processing

- Segmenting principle: message is segmented into meaningful units rather than a continuous unit
- Spaced education principle: educational encounters that are spaced (space distribution) result in more efficient learning and improved learning retention compared with mass distribution of the educational encounters (bolus education) [29,33-35]
- Pretraining principle: people learn better when they know the names and characteristics of the main concepts
- Progressive difficulty: intrinsic cognitive load depends on the expertise of the learner in the domain (knowledge available in long-term memory), so to reduce it, the difficulties related to breaking bad news points to issues that are improved as the sessions progress (as the learner becomes more of an expert). Moreover, in our training design, we further enhanced the level of difficulty by proposing two last items (session 12) without any response options (for full space for reflection) to extend acquired skills [28]

##### Foster Generative Processing

Personalization principle: the words are presented in a conversational style, changed into first and second person with some conversational sentences

- Embodiment principle: onscreen agents display human-like gestures, movements, eye contact, and facial expressions
- Control of the pace and order of presentation principle
- The essential ideas of each training session will be reiterated at the end of the session and at the beginning of the next session to increase learning retention
- Stressing the same content repeatedly over time results in more efficient learning and improved learning retention

## Results

### Existing Protocols and Recommendations

The protocol SPIKES and recommendations from the World Health Organization and the Haute Autorité de Santé in France were some examples examined as recommendations on delivering bad news [1,8-11,36,37]. The Medical Communication Competence Scale, the Communication Assessment Tool, and the Frankfurt Observer Communication Checklist [38-40] were examined as patient-physician communication assessment instruments.

### Semistructured Interviews With Patients and Physicians

Semistructured interviews were designed to allow patients and physicians to describe their own experiences with breaking bad news, while addressing their understanding and emotions in these situations and how they manage interpersonal relationships. The main topics of the open-ended interview guides are presented in Textbox 1.

**Main topics included in the open-ended interview guides for patients and physicians**

**Patients**

1. Factual description of delivering bad news about disease
2. Cognition (eg, *questioning, anticipation, projection into the future)*
3. Emotion, behavior around the delivery of bad news
4. Interpersonal relationships (eg, *with families and work colleagues)*
5. Feelings and potential for improvement

**Physicians**

1. Factual description of delivering bad news about disease
2. Cognition (eg, *difficulties, examples of experiences, self-assessment*)
3. Emotion, behavior around the delivery of bad news
4. Interpersonal relationships (eg, *with patients and colleagues*)

### Thematic Analysis Grids

For patient interviews, 3 categories were retrieved from the thematic analysis (Multimedia Appendix 1):

- The time of delivering bad news: reaction to the diagnosis (awareness of the disease, uncertainties surrounding the disease, psychological reactions), illness representation, and evolution of interpersonal relationships (with work colleagues, the healthcare team, family, and relatives)
- Care pathway and medical care: period after the breaking of bad news, with medical care after the diagnosis (description, questions and doubts of the patient, psychological reactions, interpersonal relation)
- Life with the disease (how care is delivered, physical and psychological impacts of the disease, adjustment to the illness)

For physician interviews, 8 categories were retrieved from the thematic analysis (Multimedia Appendix 2):

- Elements that might be taken into account for breaking the bad news
- Difficulties experienced by the physician at the time of delivering bad news
- Physician’s training in delivering bad news
- Information provided by the physician about medical treatments and overall disease management
- Specific characteristics of the disease
- Physician-patient relationship
- Physician’s perception of their role
- Physician’s emotional experience

### Construction of the Good Practices Communication Framework List by Experts

In total, 70 dimensions or specific issues related to breaking bad news were selected and structured. The good practices communication framework list consisted of 8 main domains, each domain consisting of dimensions constituting items to be developed in BRADNET (the number in brackets refers to the number of dimensions for each domain):

1. Opening (3): general questioning about the experience of breaking bad news, such as the kind of bad news delivered by the physician
2. Preparing for the consultation wherein bad news will be delivered (3): physicians are asked to consider how they prepare for a consultation in which they have to deliver bad news (eg, ensuring an enabling environment, asking patients to be accompanied by relatives)
3. Communication techniques (22): devoted to attitudes and behavior that can improve the quality of the physician-patient relationship (eg, introduce oneself, take time, respect silence, and ask open-ended questions)
4. Consultation content (19): the physician is asked to question the kind of the information that should be sought (eg, the patient's concerns) and transmitted (depending on the patient's beliefs or health skills) and how the patient can be supported (eg, by mentioning the future and life with the disease)
5. Attention (5): the patient’s verbal and nonverbal attitudes, emotional difficulties, and cognitive limitations, to which the physician should pay attention
6. Physician emotional management (3): physicians are asked to question their emotions during a consultation wherein bad news is delivered, their behaviors and attitudes that betray their emotions, and how they cope with these emotions
7. Shared decision making (12): how physicians can develop a relationship with the patient, during which the patient has a role of care partner with whom decisions are made
8. Relationship between the physician and the medical team (3): questions of transmitting information regarding the patient or care of the patient to other healthcare professionals

With the good practices communication framework list thus defined, the final intervention could be developed.

### BRADNET Items Development

Experts constructed 70 items based on the good practices communication framework list. Questions were built to foster reflection on how each physician delivered bad news in the past to anchor messages introducing recommended communication behaviors related to the physician’s practice. The answering modalities of an item were a particular communication behavior in a specific situation (see items from domains 2, 4, 6, 7, 8 in Multimedia Appendix 3) or questions assessing the frequency of a specific behavior (see items from domains 3 and 5 in Multimedia Appendix 3).

### BRADNET Refinement

As a result of the first refinement phase, besides extensive rewording, adaptation of the content and message adjustments, an additional dimension “the severity of the diagnosis also depends on the patient and their needs” was added to the domain “Consultation content.” The item generated highlights that the severity of the diagnosis depends on individual perceptions and that the impact of delivering bad news on individual perceptions should not be underestimated, even if it may seem unimportant. Finally, the Web-based self-assessment tool to practice delivering bad news, BRADNET, was composed of 71 items (ES, LR, ACR).

The analysis of the verbal material resulting from the think-aloud approach allowed for classifying the elements of the verbatim into 8 points:

1. Reflections on the content of the items: physicians verbally indicated their degree of support with each of the items: complete support, confirming what is said in the questions, propositions or messages; partial support; or disagree with all or some of the items.
2. Items analysis: physicians show hesitations, misunderstandings, want to correct text, criticize the item, or simply make remarks.
3. Analysis of their own practice: physicians question their own practice and how to improve it in terms of the questions posed by the tool.
4. Reactions to the items: brings together all the psychological defensive processes observable in the discourse of physicians when they are confronted with the content of the formative self-assessment tool: processes of resistance, distancing, reassurance, conflicts or opposition, identification, humor, doubt, and associations.
5. Factual description of the practice: factual aspects of the practice of medicine mentioned by the participants. These are comments without reflection and conceptualization; the participants listed what they do without associating it with any meaning or representations.
6. Associated thoughts: thoughts not directly related to the tool but derived from its analysis.
7. Global assessment and suggestions for improvement.
8. Prospects for future use: based on their own experience and perspectives from the initial training, participants reflect on proposing a relevant implementation of the tool.

Following this step, the items were again extensively refined to make them more useful to the target audience (ES, LM, MP).

Examples of BRADNET items are shown in Multimedia Appendix 3.

### BRADNET Learning Sessions

In total, 12 formative self-assessment sessions were constructed (8 sessions with 6 items, 2 sessions with 7 items, 1 session with 5 items, and 1 session with 4 items). Five sessions included items from 4 main domains, 4 sessions from 4 main domains, and 3 sessions from 5 main domains. Session durations were estimated at 10 to 15 minutes*.* The proposed length of the total self-assessment was 45 days to maximize the retention of the messages and to start applying changes progressively during the self-assessment. Two sessions a week seemed to be manageable for physicians with high workloads and time pressures.

### BRADNET Web-Based Platform Development

#### Description of the Web Platform

The interface will consist of a modal window of connection, allowing either the inscription on the website and the creation of a user account or a login to access the contents of the website. These identification data will be used to track the user's progress throughout the sessions and the items included in each session.

At first use, a welcome message will introduce the purpose and flow of the formative self-assessment program. During subsequent uses, this welcome message will recall where the user is in the program, focus on key elements of the previous session, and offer the ability to resume the course of the program where the user left off.

Each session will offer 4-6 items consisting of a question, predefined response modes or free input fields, and a message offering information or reinforcements on the attitudes and behaviors that may be appropriate to the situations encountered by the user in his or her practice. The sequence will be implemented as follows: the question appears first, then after 5 seconds, the response proposals or the free text field that was initially blurred appears on the screen under the question. Once the user has provided a response, a “continue” button appears and allows the user to display the message related to the question asked. This message is accompanied by a button to go to the next item but remains inoperative for 15 seconds (grayed out), allowing the user to take the time to read the message before proceeding to the next step.

At the end of a session, the user is thanked and is encouraged to return to the website in a few days to partake of the next session. The user will be offered the possibility to print the session in a pdf format.

During the use of the application, the following information is collected: the dates and times of connection, the time spent on each page or before the click to move to a next step in the program (eg, time spent displaying the message of an item), the overall time during each login session, and answers given.

The Web application was initially configured using a content management system (CMS) specifically developed for the purposes of the study and the collection of information mentioned above. CMS must allow for 1) setting up the greeting messages, the number and contents of the sessions, the expected durations between the sessions, the time of presentation of the messages, the possibility of sending a reminder by email the day before or the same day of a session, etc, and 2) accessing the data of use of the tool. All collected data will be managed by a website administrator with strict confidentiality rules.

Consistency of BRADNET features with theoretical learning principles is described in Table 1.

**Table 1:** Description of the BRADNET features and theoretical learning principles.

Theoretical learning principles			BRADNET features
Multimedia principle			Presentation of words and pictures
**Reduction of extraneous cognitive load**			
	Coherence principle [1,2]	Expressing questions and messages in a neutral and simple language	
	Signaling principle (reduction of extraneous cognitive load)	Highlighting key ideas of the items messages Provide take-home messages at the end of each session	
	Spatial contiguity principal	Combining written text and pictures or illustrations in the items messages (eg, a physician looking at the computer screen) when appropriate	
**Manage essential processing**			
	Pretraining principle	Presentation of the formative self-assessment tool at the beginning of the training: description of the 8 domains addressed, number of sessions and items by sessions and approximate time needed to complete one session	
	Segmenting principle Spaced education principle (Allow for starting to apply changes progressively during the self-assessment and to become aware of the content of the message, implement it, and ponder the messages)	Learning sessions organization: segmentation in 12 spaced sessions	
	Limited capacity assumption	Short sessions	
	Progressive difficulty	Learning sessions organization: progression of difficulty Practical situations and examples	
**Foster generative processing**			
	Personalization principle	Use of the first and second person conversational style	
	Adaptation of the tool to health care professionals and daily practice	Refinement and reformulation of items and messages by healthcare professionals	
	Control of the pace principle	Presentation of the session item by item (one screen for the questions and answering modalities and one screen for the message and key words) with a “continue” button at the bottom right side of the screen.	
	Reinforcement of long-term memory	Recall of the key messages at the beginning of the next session	
**Active learning, engagement of the learner**			
	Development of understanding, abilities, motivation, and self-regulation [3]	Formative self-assessment Reflection on one’s practice Open questions Messages provide no judgment on practice or behavior Refractory period between the screens (whatever the judgment of rate of learning [4], BRADNET encourages physicians to take the time to read the message before proceeding to the next step by means of a 15-second blurred “continue” button)	
Support of the physician during implementation			Session printed in a pdf format

## Discussion

### Principal Findings

The formative self-assessment tool, BReaking bAD NEws Tool (BRADNET), for physicians to practice breaking bad news to patients, contains 71 items, each including a question, response options, and a corresponding message. Questions are as important as messages. These 71 items were divided into 8 domains practiced with 12 formative self-assessment sessions.

A Web-based tool has the potential to reach a large number of physicians at low cost and thus ensure maximal dissemination. Indeed, a Web-based communication skills training that can be followed at any time is more accessible than classroom training. It can also reach physicians with weak motivation to follow intensive face-to-face training. Web-based teaching has been successfully developed for a variety of medical training domains. The advantages include accommodating different learning styles, a self-paced mode, and the use of computer-adapted technology. This approach allows for multiplying different educational supports and is consistent with recent teaching approaches [43-45].

Research on clinical communication training demonstrating efficacy and sustained effects is sparse [46,47]; teaching methods involve mainly role playing, simulated patients or objectively structured clinical exams. Few interventions have included virtual humans or computer-based interactions [13,48-50]. Kron et al studied the impact of virtual humans on the capacity for interacting using a wide range of communication behaviors to train students’ communication skills. Like BRADNET, the training encourages reflection during and after their interaction with virtual humans, is interactive, and encourages active learning and practice. Trained students displayed improved communication performance on the objective structured clinical exam and attitudes and experiences. They valued the ability of the program to provide immediate feedback, teach nonverbal communication skills, and prepare them for emotion-charged patient encounters, all themes addressed in BRADNET [48]. In another Web-based intervention, students reviewed a video presenting history-taking, breaking bad news, and shared decision making. They identified and marked key events and attached written reflections. Critical self-reflection and active engagement of the students in their own learning were the main learning principles [49]. Finally, a computer-based test measuring medical students’ communication skills in the field of shared decision making tested factual knowledge and applied knowledge by presenting patient vignettes. The control group scored significantly lower than the intervention one [13].

BRADNET was built to take into account physicians’ time constraints and to optimize communication skills learning. It contains several principles to lessen physicians’ cognitive load while attending the training sessions (segmenting principle, coherence principle, dual channel assumption, temporal contiguity principle, signaling principle, spaced education principle). Learning sessions are short, spaced, and organized to increase difficulty and reflection over the training to allow learners to construct schemas when they connect new information to the things they already know and further decrease cognitive load. Segmentation of content into spaced sessions is important to deal with time constraints, to enable the learner to become aware of the content of the message and implement it, and to return to the messages.

The originality of our project lies in the development of a generic tool suitable for different chronic diseases. So, far, guidelines about delivering bad news have been developed mainly to deliver a diagnosis of cancer, but every chronic disease, irreversible condition, or striking event can have a major effect on a patient’s life. How bad news is communicated can have consequences that may affect patients, sometimes definitively. It likely affects the patient–health care provider relationship, therapeutic alliance, adhesion to care, and patients’ subsequent adjustment to the disease [51]. Consequences of a chronic disease not only depend on its medical severity but also on individual perceptions, needs, personality, social environment, education, beliefs, and culture. Proposing personalized support and being aware of the unique nature of the physician-patient relationship is important, whatever the chronic disease. Even if different diseases have different consequences and representations, the impact of the quality of breaking bad news plays a critical role.

**Figure 3:**
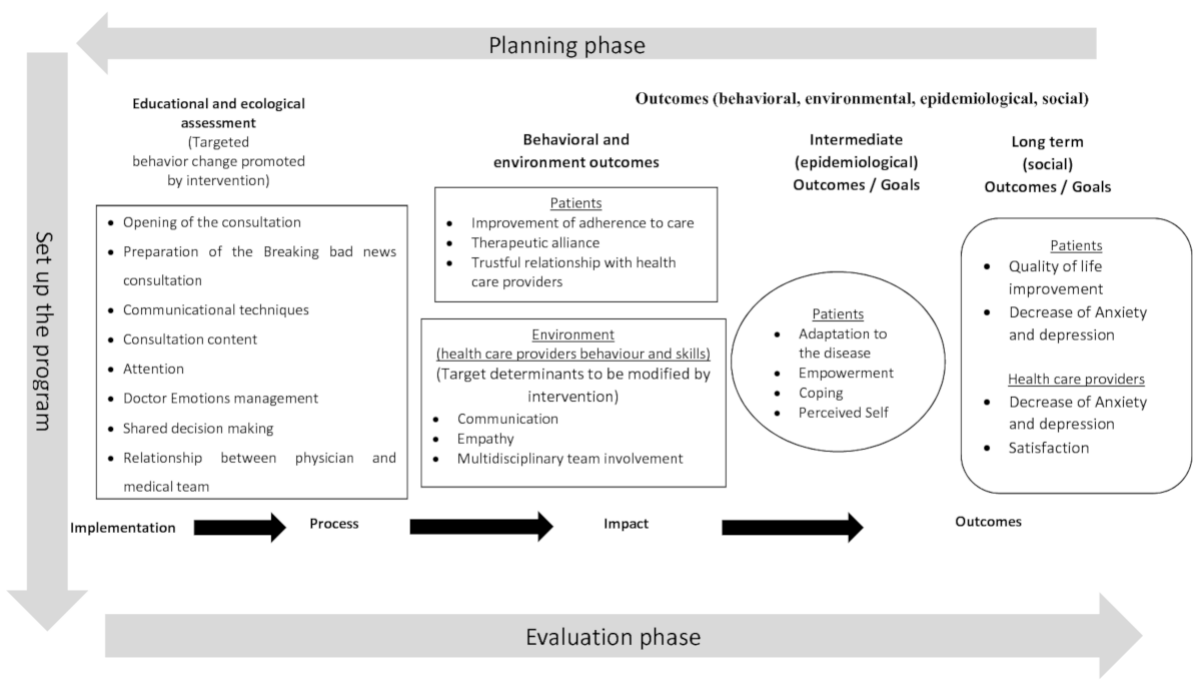
Theoretical framework.

The content of BRADNET was developed by iterative processes that integrated theoretical aspects, interviews, and expert input. By collecting broad and deep information from various sources and by using a refinement step, we tried to increase the accuracy and validity of the BRADNET content [52,53]. To design the study to assess the impact of BRADNET on healthcare professional skills and behaviors and on patients, we propose a logic model (Figure 3) based on the PRECEDE-PROCEED model (PPM) to replace the intervention in a context of evaluation.

In the PPM model, PRECEDE means Predisposing, Reinforcing, and Enabling Constructs in Educational/environmental Diagnosis and Evaluation and PROCEED means Policy Regulatory, and Organizational Constructs in Educational and Environmental Development. In this planning model, all aspects of a person’s environment are considered potential intervention targets, as are the person’s skills and behavior. The plan starts from the end goal of producing objectives and intermediate objectives, to developing a plan to achieve the objectives defined and then implement and evaluate the intervention [54]. Assessment of BRADNET will include measurements of the different patient outcomes: long-term outcomes (eg, quality of life, depression), intermediate outcomes (eg, coping, empowerment), behavioral and environmental outcomes (eg, adherence to care, relationship with health care provider), and processes (judgment or satisfaction of the physicians with the different BRADNET features, consistent with learning principles such as session durations, recall of the key messages or emphasis on key ideas).

A potential limitation of this study was the absence of specialists in the science of education involved the development of BRADNET. However, FG, LM, ACR, RV, and ES are academic professionals and are thus experienced in teaching, and ACR also teaches in a graduate diploma in therapeutic education or self-management programs. Moreover, formative self-assessment adopts principles of psychologic interviews: benevolence, understanding, and bringing knowledge without imposing it. Creating a space for reflection by giving the opportunity to initiate change is one approach to behavioral change.

### Conclusion

In conclusion, most courses for training on the disclosure of a bad news use either classical classroom training sessions on communication skills training courses [11,55-58] or classical training plus standardized patient intervention [59-63]. Our approach provides a distance-learning self-training tool that is not expensive and more manageable and less time-consuming for physicians with often overwhelming schedules. BRADNET was built to take into account physicians’ time constraints. The Web-based tool will further be tested in a randomized controlled trial with patients.

### Practical Implications

The BRADNET intervention, a simple Web-based intervention, is expected to meet physicians’ needs when breaking bad news. In France, only a few physicians have been trained and there is a need for training and personal self-reflection related to the delivery of bad news. BRADNET could be used to complete a previous training or be considered as a basic training. Improving the delivery of bad news could improve patients’ adaptation to diseases and therapeutic alliances and decrease their anxiety and depression.

## Appendix

Multimedia Appendix 1 Supplementary table: Breaking Bad News Reading Grid for patient.

Multimedia Appendix 2 Supplementary table: Breaking bad news Reading Grid for physicians.

Multimedia Appendix 3 Supplementary table: Examples of 8 items, one for each of the 8 main domains of BRADNET.

## Abbreviations

CMS: content management system
IM: intervention mapping
PPM: PRECEDE-PROCEED model
SPIKES: Setting-up, Perception, Invitation, Knowledge, Emotion, Strategy
BRADNET: BReaking bAD NEws Tool
