# Supplementary material for: Development of a Web-Based Formative Self-Assessment Tool for Physicians to Practice Breaking Bad News (BRADNET)
Source: JMIR Med Educ. 2018 Jul 19;4(2):e17. doi: 10.2196/mededu.9551 (PMC6072977; doi:10.2196/mededu.9551)
Supplement: Multimedia Appendix 1 [file mededu_v4i2e17_app1.pdf]

Breaking Bad News: Reading grid for patients

This reading grid is composed of three main categories:

*(1) the category"**Breaking bad news**" explores the communication around breaking bad news and the relational sphere of the patient linked with breaking bad news,*

*(2) the category"**Care pathway and medical care**" touches on medical examinations and medical treatments surrounding the breaking of bad news,*

*and (3) the category **"living with the disease"** talks about the daily life with a disease.*

1. **Breaking bad news: communication and relationship**
    a. **First acknowledgment of the disease:** an event or a symptom will trigger in the patient the feeling of being sick.
    b. **Questions and doubts of the patient**: every question and misunderstanding of the patient when discovering the disease.
    c. **Psychological reactions**: feelings and coping strategies around breaking bad news.
    d. **Interpersonal relations**: Every interpersonal relation upon breaking bad news (it can be the social support after the breaking bad news or also the emotional reactions of the close ones.)
        i. **Patient and work colleagues**
        ii. **Patient and medical staff**
        iii. **Patient and close friends and family**
            1. **Communication on breaking bad news:** for example, the patient talks to family and friends for the first time about the disease
            2. **Emotional reactions of the family**
            3. **Social support of the family**
            4. **Reactions of the patient to the social support**
        iv. **Patient and physician**
            1. **Relationship between the patient and physician**
            2. **Description of the breaking bad news**
    e. **Illness representation during breaking bad news**
    f. **Symptoms** (each of the symptoms linked to the illness and breaking bad news)

2. **<u>Care pathway and medical care:</u>** this domain concerns the period after the breaking of bad news, with the medical care after the diagnosis. It relates to the patient's reactions during the care pathway but also the questions and relations with relatives and medical staff, always during this specific time of care pathway after the diagnosis.

    a. **Description**

    b. **Questions and doubts of the patient**

    c. **Psychological reactions**

    d. **Interpersonal relation**

        i. **Patient and work colleagues**

        ii. **Patient and medical staff**

        iii. **Patient and close ones (friends and family)**

            1. **Emotional reactions of the family**

            2. **Social support of the family**

            3. **Reactions of the patient about the social support**

        iv. **Patient and physician**

3. **<u>Living with the disease</u>**

    a. **Physical and psychological effects and coping strategies**

    b. **Questions and doubts of the patient**

    c. **Interpersonal relation**

        i. **Patient and work colleagues**

        ii. **Patient and close ones (friends and family)**

            1. **Emotional reactions of the family**

            2. **Social support of the family**

            3. **Reactions of the patient about the social support**

    d. **Illness representation**
