# Supplementary material for: Development of a Web-Based Formative Self-Assessment Tool for Physicians to Practice Breaking Bad News (BRADNET)
Source: JMIR Med Educ. 2018 Jul 19;4(2):e17. doi: 10.2196/mededu.9551 (PMC6072977; doi:10.2196/mededu.9551)
Supplement: Multimedia Appendix 2 [file mededu_v4i2e17_app2.pdf]

Breaking bad news: Reading grid for physicians

1. **Determining factors for breaking bad news:** includes everything that can influence how to break bad news to a patient.

    a. **The perception by the physician of the expectations of the patient**.
    b. **The patient profile**: It can be age, personality, everything that can explain why a physician breaks bad news in different ways.
    c. **The concern for accuracy:** refers to the physician's posture. Some physicians who make announcements prefer to wait some time before announcing an illness, to be able to confirm the established diagnosis (wait until all the results of examinations are transcribed in the medical file, because announcing a diagnosis is possible only all the evidence for the diagnosis is available).
    d. **Physician's strategy:** the physician's choice according to the situation encountered: to select the information, or to meet the patient with the family. This dimension also takes into account the strategies and communication techniques as well as the posture of the physician.

2. **Difficulties encountered by the physician**: all the difficulties that the physician encounters at the time of the announcement.

    a. **Analysis of the psychological reactions of the patient**: the physician tries to detect the feelings of the patient at the time of the announcement.
    b. **Need for patient feedback:** the physician feels the need for feedback from the patient, whether it is an active and voluntary initiative or not; there is a need for reassurance from the physician on his practice. In this category we find everything that refers to the patient's feedback and also the physician's strategies to verifying the patient's understanding of the information delivered.
    c. **Organizational factors:** external factors that cause difficulties to the physician in the context of the announcement. It can be the time constraints, the means (human, in particular) available and those that can affect the announcement to the patient.
    d. **Communication with the patient:** Difficulties by the physician to evoke certain subjects or to be well understood by the patient.
    e. **Perception of the patient, of his knowledge and beliefs:** This is physician's perception about the patient's knowledge, about the illness and its sources of information. This category also refers to the patient's beliefs about the disease that may or may not be a brake for the physician in the announcement and the follow-up.

3. **Physician's training at the announcement:** The training (or not) of the physician at the announcement: courses during medical studies or practice on the field? This part involves the following:

    a. **Curriculum:** the announcement in medical studies, physician's training in the announcement etc.
    b. **In the field:** training for the announcement in the field (ie, during internships etc.)

4. **Information delivered by the physician**: includes all the information and data provided by the physician to the patient. This information concerns the disease and the treatments.

    a. **On the disease:** the physician gives explanations to the patient about the illness, its consequences, its origin, etc.
    b. **On treatment and follow-up:** after talking about the disease, the physician gives explanations (or not) about available treatments and consequences of treatments such as side effects. This category also takes into account the information relating to the follow-up of the patient (treatments during the follow-up, medical consultations, recommended lifestyle, etc.).

5. **Disease specificity**: the physician gives explanations about the specifics of the disease.

    a. **Comparison with other diseases**
    b. **Description and perception of the disease by the** physician

6. **Interpersonal relation:** surrounding breaking bad news, this main category talks about the relation between a physician and the patient, with the medical team and with the patient's family.

    a. **Physician and patient**

        i. **Therapeutic alliance**
        ii. **Trust**
        iii. **Conflict situation**

    b. **Physician and medical team**

        i. **Consistency:** to act and care every member of the medical team in the same way.
        ii. **Relay:** when the physician needs a relay for complicated situations they cannot handle themselves.
        iii. **Representations of the hospital settings:** physician's perception about the job and workplace.

    c. **Physician and patient's family**.

7. **Physician's perception about the job and function.**

    a. **Informative role**
    b. **technicality/to care/to cure**

8. **Emotional involvment and emotional reactions of the physician.**

    a. **Emotional reactions**
    b. **Frustration**: lack of time, professional boundaries
