# Supplementary material for: Development of a Web-Based Formative Self-Assessment Tool for Physicians to Practice Breaking Bad News (BRADNET)
Source: JMIR Med Educ. 2018 Jul 19;4(2):e17. doi: 10.2196/mededu.9551 (PMC6072977; doi:10.2196/mededu.9551)
Supplement: Multimedia Appendix 3 [file mededu_v4i2e17_app3.pdf]

Supplemental table 3: Examples of 8 items, one for each of the 8 main domains of BRADNET

**Domain 1**	Opening
Dimension	Be aware of the importance of delivering bad news for the patient
Item question	In delivering bad news about chronic disease, as time goes on, are you always aware of the impact the diagnosis can have on the person?<br>a) Yes<br>b) No
Item message	It becomes trivial for the physician to give the news of an illness. According to one estimate, over the course of a career, an oncologist will break bad news to patients about 20,000 times, from the first shocking facts of the diagnosis to the news that death is near. However, it must be remembered that under all circumstances, it is often a unique event for the patient, one that will probably bring many important changes in their life.
**Domain 2**	Preparing for the delivering bad news consultation
Dimension	Sustained by a physical environment
Item question	What is for you an enabling environment for delivering bad news?<br>a) An adapted and a quiet place (without possibility of intrusion)<br>b) A neutral place such as a unique place (no associated memory for the patient)<br>c) Physically at the same level as the patient (not remain standing if the patient is lying down)<br>d) No over the phone<br>e) Avoid having bulky furniture or other objects between you and the patient<br>f) If necessary, be able to propose handkerchiefs or glass of water
Item message	However, it must be remembered that in effect, it is sometimes impossible, sometimes difficult and rarely simple to have an ideal place. You can only try to follow the recommendations mainly developed for the context of cancer.
**Domain 3**	Communication techniques
Dimension	Take time
Item question	Are you spending more time during a consultation when you have to break bad news?<br>a) Always<br>b) Often<br>c) Rarely
Item message	Even if your time is limited, taking the necessary time allows for delivering the information gradually, so information on the news will be better understood by the patient.

**Domain 4**	Consultation content
Dimension	Ensure that any questions the patient might have are answered.
Item question	How do you ensure that the patient has received information about any

	issues important to them?<br>a) I ask them if they would have liked more details on…<br>b) I ask them to reflect on…<br>c) I summarize the situation<br>d) Other
Item message	Just before you finish the interview, it is important to ensure that any questions the patient might have are answered. The interview should conclude with a summary of the main points that you discussed.
**Domain 5**	Attention
Dimension	Pay attention to how much information the patient is ready to receive.
Item question	Do you look for how much information the patient is ready to receive during the consultation?<br>a) Usually<br>b) Rarely
Item message	It is recommended to always ask the patient if they are ready to receive new information. For example, immediately after patients are informed of a diagnosis, it would be pointless to offer an exhaustive and precise list of all therapeutic options. It may be appropriate to arrange a future appointment to discuss treatment options.
**Domain 6**	Physician emotional management
Dimension	During the consultation: pay attention to self-body language
Item question	Do you sometimes have the following attitudes during the consultation?<br>a) Restless<br>b) In a hurry<br>c) Arms crossed<br>d) Check the time<br>e) Yawn<br>f) Reply to your phone messages<br>g) Show signs of annoyance<br>h) Speak quickly<br>i) Not raise your eyes from your computer or your records<br>j) Answer the phone<br>k) Manipulate your pen<br>l) Other
Item message	Non-verbal communication and self-body language are perceived by patients. This is a means of strengthening the quality of the therapeutic relationship.
**Domain 7**	Shared decision making
Dimension	Talk with the patient about the different treatment options to help manage the condition
Item question	What specific information do you tell your patient about treatments?<br>a) I point out that the treatment has to be taken for the rest of life, even if it’s tiring<br>b) I point out that the treatment has to be taken every day<br>c) I talk about the protocol (frequency of re-evaluations, hospitalizations, number of treatment courses etc.)<br>d) I explain how each drug works
Item message	It is essential that the patient clearly understand that the medical treatment needs to be taken regularly over a long period of time.<br>Therefore, the patient should have a realistic picture of what they can

	reasonably expect in terms of regular medical follow-up etc.
**Domain 8**	Relation between the physician and the medical team
Dimension	Pass the task of breaking bad news on to other healthcare providers
Item question	With a patient, when you have reached your own personal limits (e.g., when the patient is young or a child), how do you pass the task of breaking bad news on to other healthcare providers?<br>a) I ask another physician from the medical service to break bad news<br>b) I break bad news with another physician<br>c) I break bad news with a nurse or a midwife<br>d) I make sure that a nurse speaks again about the bad news with the patient after the consultation<br>e) Other
Item message	Being supported by non-physician primary healthcare professionals is sometimes appropriate. Besides, physicians should learn, without feeling guilty, to pass on the task to other healthcare providers.
